# Primary Prophylaxis to Prevent the Development of Hepatic Encephalopathy in Cirrhotic Patients with Acute Variceal Bleeding

**DOI:** 10.1155/2018/3015891

**Published:** 2018-07-10

**Authors:** Fátima Higuera-de-la-Tijera, Alfredo I. Servín-Caamaño, Francisco Salas-Gordillo, José L. Pérez-Hernández, Juan M. Abdo-Francis, Jaime Camacho-Aguilera, Sai N. Alla, Fiacro Jiménez-Ponce

**Affiliations:** ^1^Gastroenterology and Hepatology Department, Mexico's General Hospital, Mexico City 06726, Mexico; ^2^Internal Medicine Department, Mexico's General Hospital, Mexico City 06726, Mexico; ^3^Research Department, Chief of the Medical Direction of “Instituto de Seguridad y Servicios Sociales de los Trabajadores del Estado” (ISSSTE), Mexico City 14050, Mexico; ^4^Cognitive Science A.C., Mexico City 10700, Mexico

## Abstract

**Background and Aim:**

Variceal bleeding is the second most important precipitating factor related to the development of episodic hepatic encephalopathy; but to date there are no recommendations to prevent this complication. The aim of this study was to compare if primary prophylaxis with lactulose or L-ornithine L-aspartate or rifaximin, in cirrhotic patients with variceal bleeding, is better than placebo for avoiding the development of hepatic encephalopathy.

**Methods:**

A randomized, double-blind, placebo-controlled clinical trial (ClinicalTrials.gov identifier: NCT02158182) which included cirrhotic patients with variceal bleeding, without minimal or clinical hepatic encephalopathy at admission.

**Findings:**

87 patients were randomized to one of four groups. The basal characteristics were similar between groups. Comparatively with placebo, the frequency with regard to the development of hepatic encephalopathy was as follows: lactulose (54.5% versus 27.3%; OR = 0.3, 95% CI 0.09-1.0;* P* = 0.06); L-ornithine L-aspartate (54.5% versus 22.7%, OR = 0.2, 95% CI 0.06-0.88;* P* = 0.03); rifaximin (54.5% versus 23.8%; OR = 0.3, 95% CI 0.07-0.9;* P* = 0.04). There was no significant difference between the three groups receiving any antiammonium drug (*P* = 0.94). In the group receiving lactulose, 59.1% had diarrhea, and 45.5% had abdominal discomfort, bloating, and flatulence. Two patients (10%) treated with lactulose and a patient (4.5%) in the placebo group developed spontaneous bacterial peritonitis due to* E. coli*; one of them died due to recurrent variceal bleeding. There were no other adverse effects.

**Conclusions:**

Antiammonium drugs, particularly L-ornithine L-aspartate and rifaximin, proved to be effective in preventing the development of hepatic encephalopathy in those cirrhotic patients with variceal bleeding.

## 1. Introduction

Hepatic encephalopathy (HE) is a neurological disorder caused by the accumulation of toxic substances in the blood due to the inability of liver to perform its detoxification functions [1]. Ammonium plays an important role in the pathophysiology of this disorder, and even the currently available treatments for HE are designed to reduce the production and intestinal absorption of ammonium or to promote the metabolism thereof in extrahepatic tissues. Treatments that have proven to be effective in both minimal HE (MHE), detected through changes in neuropsychometric tests, and in overt HE (OHE) include nonabsorbable disaccharides such as lactulose, antibiotic that acts in intestinal lumen such as rifaximin, and drugs favoring extrahepatic metabolism of ammonium such as L-ornithine L-aspartate (LOLA) [2–5].

It is well known that the development of HE deteriorates the cognitive function in cirrhotic patients and also predisposes to risks such as increased frequency of falls. This cognitive impairment has a significant negative impact on the quality of life of these patients [6, 7].

Severe cases of HE can lead to coma and death [7]. In patients with acute on chronic liver failure it has been shown that the development of HE independently predicts higher mortality [8, 9]. In this clinical context, studies show extremely high mortality in patients who develop HE with cerebral edema [10]. In 2009, in a period of one year, Fichet reported a 54% mortality-rate in patients with severe HE admitted in intensive care units [11].

Acute variceal bleeding (AVB) occurs in 25 to 30% of patients with cirrhosis [12]. The development of HE in patients with AVB is a well-known complication and the incidence of this complication ranges from 16.9 to 40% [13, 14]. The absorption of toxic products such as ammonium is the main mechanism involved in the development of HE after an episode of AVB. In order to favor the elimination of blood from the gastrointestinal tract various treatment strategies had been used such as bulking enemas and intestinal irrigation with mannitol [15–19]. However, currently these therapies are not recommended and therefore not used routinely.

To date, only two nonblinded studies have evaluated oral administration of lactulose versus placebo, demonstrating that lactulose is an effective therapy to prevent the development of HE after an AVB [13, 14].

The aim of this study was to compare whether the clinical effect of primary prophylaxis with lactulose or LOLA or rifaximin in cirrhotic patients with AVB is better than placebo for preventing the development of HE.

## 2. Methods

A randomized, double-blinded, controlled clinical trial (ClinicalTrials.gov number NCT02158182) was performed at “*Hospital General de México*”. The selection criteria included cirrhotic patients, both genders, admitted to hospital for AVB, without MHE assessed by the Psychometric Hepatic Encephalopathy Score (PHES) and critical flicker frequency (CFF) or OHE according to West-Haven criteria [20]. Exclusion criteria were patients under 18 years and over 65 years; patients with any type of dementia or any manifestation of neurological disease; patients with any type of bacterial infection at admission; patients who were receiving secondary prophylaxis for spontaneous bacterial peritonitis (SBP) with norfloxacin or another antibiotic because we thought it could be a bias for our investigation since infections are the main precipitating factor of episodic HE; patients with a previous diagnosis of MHE or OHE and who were receiving treatment with specific therapies for HE; patients whose aetiology of AVB and portal hypertension were distinct from cirrhosis; patients with serum creatinine ≥ 2.0 mg/dL or previously diagnosed with chronic renal failure; patients treated in the previous 6 months with any of the drugs used in this clinical trial. Patients with severe AVB who were hemodynamically unstable or who required orotracheal intubation at admission were not eligible for this trial. Patients who withdrawn their informed consent to participate in the study and who have not completed at least one evaluation after treatment were eliminated.

### 2.1. Sample Size

We used the formula for contrasting hypotheses of two proportions. We considered a one-sided level of significance of 5% (*α* = 0.05) hypothesizing that any of the prophylactic manoeuvres employed would be superior to placebo and also considering noninferiority between the antiammonium therapies. We considered a statistical power of 80% (1-*β* = 0.80) acceptable. As at the time of design this study there were no previous clinical trials about preventing the development of HE in cirrhotic patients with AVB treated with rifaximin or with LOLA, we based our sample size calculation on the assumption that an acceptable difference would be to find 40% less development of HE in those treated with lactulose or with rifaximin or with LOLA, each group by itself compared to the placebo group. With these data the sample size was 18 patients per group but we considered an additional 20%, because of possible losses (4 patients per group). Hence the sample size of 22 patients per group was assigned.

### 2.2. Definition of Terms

*Primary Prophylaxis*. It is also called “primary prevention” which is defined as those measures directed to prevent a condition or disease [21]. For this study, primary prophylaxis strategies were the administration of lactulose or rifaximin or LOLA, aimed at preventing the development of OHE in cirrhotic patients admitted for AVB.

*Overt Hepatic Encephalopathy (OHE)*. It was defined according to the American Association for the Study of Liver Diseases (AASLD) and European Association for the Study of the Liver (EASL) guideline as “brain dysfunction caused by liver insufficiency and/or portosystemic shunting, and manifests as a wide spectrum of neurological/psychiatric abnormalities ranging from mild clinical alterations to coma”. OHE can encompass a wide spectrum of mental and motor disorders and may arise episodically over a period of hours of days in a previously stable patient [22]. Clinically we assessed patients daily through the West-Haven criteria [20]. Clinical diagnosis was performed by carrying out a systemic neurological exploration by a blinded expert neurologist.

*Minimal Hepatic Encephalopathy (MHE)*. It means absence of evident clinical manifestations, but with alterations in neuropsychometric tests, such as PHES and/or CFF [20, 22].

*Acute Variceal Bleeding (AVB)*. Cirrhotic patients were admitted to hospital for hematemesis and/or melena and demonstrated endoscopically the presence of esophageal or esophagogastric varices with active bleeding or red signs suggesting inactive, but recent bleeding. The AVB was handled specifically according to the recommendations of practice guidelines of the AASLD [19].

*Spontaneous Bacterial Peritonitis (SBP).* This diagnosis was clinically suspected in those who developed symptoms and/or signs of peritoneal irritation, but also in those who developed HE, since several cases of SBP are oligosymptomatic. In all these cases, we performed a diagnostic paracentesis to obtain a polymorphonuclear count (PMN) and ascites culture. The diagnosis was confirmed by a PMN ≥ 250 cells/mm3 in ascites and a positive ascites culture. It was first empirically treated according to the recommendations of the AASLD practical guidelines [23], and then the antibiotic treatment was adjusted according to the report of ascites culture.

### 2.3. Study Endpoints

The primary endpoint was the development of OHE in the first week (7 days) after an AVB. Secondary endpoints were as follows: The time in days for the development of OHE after an AVB; also the late occurrence of OHE on the first 28 days after an AVB; finally, the development of adverse effects during therapy. Specifically, for the group receiving rifaximin, a secondary endpoint was to verify the frequency of development of SBP in comparison with the other groups.

### 2.4. Procedure and Randomization

*Preinclusion Phase.* Informed consent was obtained for all participants in this study; medical history along with neurological examination of every patient was performed, to rule out OHE; the West-Haven criteria were applied [20]. Likewise, the mini-mental test was performed; if the result was normal, then the PHES was applied and finally the CFF was performed in order to detect those patients with MHE. Patients with OHE or MHE at admission were excluded from this study.

At admission laboratory studies were taken: urea, creatinine, sodium, chlorine, potassium, total bilirubin, albumin, hemoglobin levels, leukocytes count, platelets count, prothrombin time, and international normalized ratio (INR).

In case of ascites diagnostic paracentesis was performed for PMN count and culture; moreover urinalysis, blood and urine cultures, and chest X-ray were performed to exclude infections at admission. Endoscopy was performed within the first 12 hours after admission to determine the source of bleeding. Number of red cells units transfused, hemodynamic parameters such as cardiac frequency and mean arterial pressure at admission, and recurrence of AVB were recorded.

*Randomization.* A person was the monitor who made the black bags for containing the medications and the corresponding placebos, for all different groups, and this person did not participate in allocation or clinical evaluation of patients.

After the initial assessment by a blinded investigator, patients who met selection criteria and signed the informed consent were allocated to one of four possible groups by another investigator; also he was blinded to the drugs administered in each group; he only knew the name of the group by the letter (A, B, C, or D) but he did not know which medication corresponded to the letter of the group. Randomization was carried out using a table of random numbers considering four groups of equal size, constructed with the Epidat 3.1 statistic program (Galicia, Spain 2006), to evenly distribute patients to each treatment groups. The investigator, who allocated patients to the treatment groups, never had contact with them.

Group A was treated with lactulose (Lactulax®) orally, 30 mL every 8 hours, while patients had residual melena; then it was adjusted by increasing or reducing the dose to 10 ml every day according to the dose response to achieve two to three daily soft stools. Group B was treated with LOLA (Hepa-Merz®) intravenous infusion (500 ml of saline solution containing 10 grams of LOLA for 24 hours). Group C was treated with rifaximin (Flonorm®) administered at a standard dose of 400 mg orally every 8 hours. Group D was the control group that received all the corresponding placebos to achieve blinding of the study; patients in this group received an intravenous glucose solution of 5% for 24 hours, dextrose solution of 30 ml orally every 8 hours, adjusted equally as mentioned for lactulose dose, and 2 dextrose tablets orally every 8 hours in similar size, color, and shape to the rifaximin tablets. Groups A, B, and C also received treatment corresponding to the complementary placebos to ensure that both the investigator and the patient were blind towards the prophylaxis maneuver they were receiving. In all groups treatment duration was 7 days.

### 2.5. AVB Treatment

Hemodynamic stabilization, as well as vasopressors (octreotide or terlipressin), was administered following the recommendations of the AASLD practical guidelines [19]. Endoscopic study was conducted in the first 12 hours after admission. In case of AVB by esophageal varices band ligation was performed, and in case of AVB by gastric varices sclerotherapy was used.

### 2.6. Primary Prophylaxis of Infections

Quinolones or cephalosporins were administered during a period of 7 days according to the recommendations of practice guidelines from the AASLD [19, 23]. In group C patients, rifaximin was the only antibiotic administered, because a specific secondary endpoint in this group was to verify the frequency of development of SBP in comparison with the other groups. This group received saline solution intravenously instead of the systemic antibiotics to try blinding the study correctly.

### 2.7. Follow-Up

Patients were reassessed daily through West-Haven scale and systematic neurological exploration searching for OHE [20]. Laboratory controls were taken on day 3 and on day 7; these included urea, creatinine, sodium, chlorine, potassium, bilirubin, albumin, also hemoglobin, leukocytes count, platelets count, prothrombin time, and INR.

In case of clinical suspicion of infection development, a control diagnostic paracentesis for PMN count and ascites culture and urine and blood cultures and chest X-ray were taken.

After 7 complete days of therapy and follow-up, stable patients without additional complications were discharged for outpatient follow-up. Only those patients who required treatment due to development of OHE or any other complications stayed in hospital for management. Thereafter, all patients were reassessed every week until 28-day follow-up searching for late complications.

### 2.8. Statistical Analysis

Quantitative variables were expressed as mean and standard deviation or median and range according to their parametric or nonparametric distribution. The qualitative variables were expressed as a ratio and percentage. To compare among groups, Student's t-test or Mann–Whitney U test and one-way ANOVA with post hoc contrasts were applied using LSD test or T2 Tamhane according to the homogeneity of variances; Kruskal-Wallis, Chi-square, and Fisher's exact tests were applied as appropriate. To estimate the risk, odds ratios and their respective 95% confidence interval were calculated. For the multivariate analysis, binary logistic regression was performed. Additionally we constructed Cox regression models. Epidat 3.1 statistical package (Galicia, Spain 2006) and SPSS version 19.0 (Chicago, IL 2010) were used.

### 2.9. Ethical and Biosafety Aspects

All patients signed the written informed consent to participate in the study. This study was approved by Institutional Ethics and Investigation Committees.

## 3. Results

**Figure 1:**
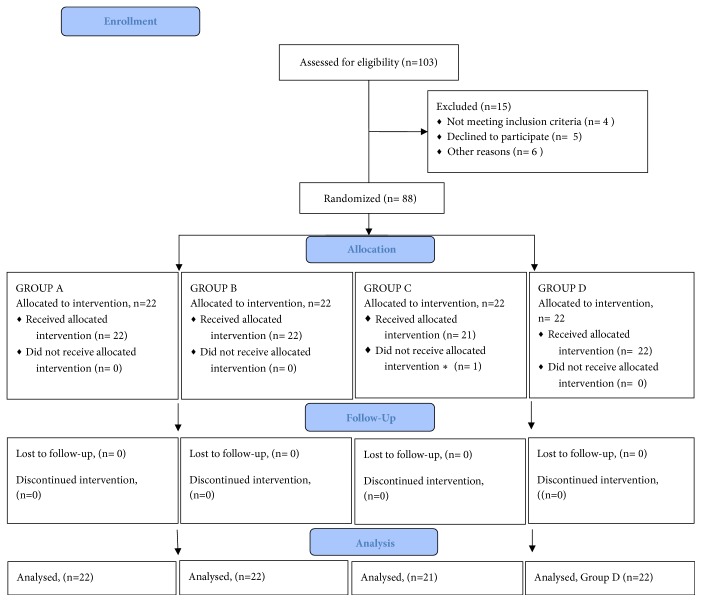
Group A was treated with lactulose orally, 30 mL every 8 hours; meanwhile it was adjusted according to the dose response to achieve two to three daily soft stools. Group B was treated with LOLA administered at a standard dose of 10 grams intravenously diluted with saline solution of 500 ml with a continuous infusion for 24 hours. Group C was treated with rifaximin administered at a standard dose of 400 mg orally every 8 hours. Group D was the control group that received all the corresponding placebos to achieve blinding of the study; patients in this group received an intravenous glucose solution of 5% for 24 hours, dextrose solution of 30 ml orally every 8 hours, and 2 dextrose tablets orally every 8 hours in similar size, color, and shape to the tablets of rifaximin. Groups A, B, and C received also the other corresponding placebos in other double-blind study. The duration of therapy was 7 days in all groups. ^*∗*^Withdrawn his informed consent.

103 cirrhotic patients were evaluated and admitted for AVB; of them 15 met some exclusion criteria (4 were older than 65 years, 3 were receiving secondary prophylaxis with norfloxacin for SBP, 3 had recent intake of alcohol, and 5 did not agree to participate). 88 patients were randomized to one of four possible groups, but one in group C finally withdrew his consent to participate in the study. See Figure 1. The baseline characteristics of patients included in the study are summarized in Table 1.

### 3.1. Development of Overt Hepatic Encephalopathy (OHE)

In the placebo group 12/22 patients (54.5%) developed OHE after the AVB episode; in the lactulose group this occurred in 6/22 patients (27.3%); in the group receiving LOLA this occurred in 5/22 patients (22.7%); and in the group receiving rifaximin this occurred in 5/21 patients (23.8%). Comparatively with placebo, the frequency regarding the development of OHE was as follows: lactulose (54.5% versus 27.3%, OR 0.3, 95% CI 0.09 to 1.1;* P* = 0.06); LOLA (54.5% versus 22.7%, OR 0.2, 95% CI 0.06 to 0.88;* P* = 0.03); rifaximin (54.5% versus 23.8%, OR 0.3, 95% CI 0.07 to 0.9;* P* = 0.04). When we compared the three groups that received antiammonium therapies, we found no significant differences between the three groups (*P* = 0.94).

The time in days from admission to the development of OHE among those who developed it was as follows: lactulose, median 2.5 (range: 2-4); LOLA, median 3 (range: 1-3); rifaximin, median 3 (range: 1-4); placebo, median 2 (range 1-4);* P = *0.88. Nobody developed OHE beyond day 4 on 28-day follow-up.

Regarding the degree of OHE according to the West-Haven criteria, the degree of OHE was more severe in those who received placebo (median 3, range 2-4) compared to those who received any antiammonium prophylactic measure: LOLA (median 1, range 1-2) (*P* = 0.04); rifaximin (median 2, range 1 to 3) (*P* = 0.05); and lactulose (median 2, range: 1 to 3) (*P* = 0.02).

### 3.2. Adverse Effects

In the group receiving lactulose 12/22 patients (54.5%) had diarrhea and required a dose reduction to 50% (15 ml orally every 8 hours); also 2/22 patients (9.1%) required reducing the dose to 10 ml every 12 hours to achieve the goal of two to three soft stools a day but without diarrhea. Moreover 10/22 (45.5%) reported bloating, abdominal discomfort, and flatulence.

Two patients (9.1%) in the lactulose group developed SBP secondary to* E. coli*; one died on day 10 of follow-up as a consequence of the recurrence of AVB. One patient (4.5%) in the placebo group also developed SBP (*E. coli*); this person improved with adjusted antibiotic treatment according to the results of bacteriological culture.

One patient (4.8%) in rifaximin group had nausea and dyspepsia, no other adverse events were recorded in this group, and no patient in this group developed SBP at 1-month follow-up. One patient in this group died at day 15 of follow-up due to recurrence of the AVB. In the group of LOLA no adverse events or deaths were registered.

### 3.3. Comparison between Characteristics of Patients Who Developed OHE versus Those Who Did Not Develop OHE

Patients who developed OHE subsequent to the AVB episode recorded a lower mean arterial pressure on admission compared to patients who did not develop encephalopathy (64.1 ± 10.8 versus 69.6 ± 10.2;* P* = 0.02). The serum albumin was lower among patients who developed OHE versus those who did not develop OHE (2.7 ± 0.7 versus 3.2 ± 0.5;* P* < 0.0001). Prothrombin time (19.3 ± 9.3 versus 15.4 ± 3.6;* P* = 0.03) and INR (1.6 ± 0.8 versus 1.3 ± 0.3;* P* = 0.03) were longer among those who developed OHE versus those who did not develop OHE. See Table 2.

In the univariate analysis the recurrence of AVB and decompensated (Child B or C) cirrhosis were identified as predisposing factors associated with the development of OHE. On the other hand, receiving prophylaxis with some antiammonium therapy was a protective factor that prevented the development of OHE following an AVB episode. Neither the development of SBP nor the origin (esophageal or gastric) of variceal bleeding influenced the development of OHE. See Table 3. The multivariate analysis confirmed that the recurrence of AVB is the main risk factor for the development of OHE (OR = 12.1; 95% CI 3.5-42.5;* P* < 0.0001). The multivariate analysis also confirmed that receiving prophylaxis with any antiammonium therapy was a protective factor to avoid the development of OHE in cirrhotic patients following an episode of AVB (OR = 0.2; 95% CI 0.05 to 0.6;* P *= 0.006). See Table 4.

Additionally, with the variables that showed statistical significance in the univariate analysis, we constructed two distinct Cox regression models, the dependent variable was the development of OHE, and the time for occurrence of OHE was determined in days. In the first model we introduced dichotomous variables: recurrence of bleed, decompensated cirrhosis, and receiving or not an antiammonium therapy. In the second model we introduced categorical variables: recurrence of bleed, cirrhosis stratified according to Child (A, B, or C), and group of treatment (A, B, C, or D). None of the two models demonstrated statistical significance for any variable. See Table 5.

## 4. Discussion

The AVB is recognized as the second most important factor triggering episodic HE [24, 25], but to date, there are no recommendations or sufficient evidence regarding which strategies could prevent this complication.

Our study shows that primary prophylaxis with antiammonium drugs, started early in cirrhotic patients admitted due to AVB, is a strategy which is effective in avoiding the development of OHE, globally decreasing the incidence of OHE in 25.9% when compared with the placebo group; this is consistent with what other authors have previously reported, such as P. Sharma et al. [14], who in an open clinical trial compared the prophylactic effect of lactulose with placebo in preventing the development of OHE in cirrhotic patients with AVB and found a difference, between groups, of 26% in favor of the group treated with lactulose.

Our study highlights that lactulose was the antiammonium drug that did not strictly reach statistical significance to prevent the development of OHE in cirrhotic patients with AVB compared with placebo (*P* = 0.06). However, when its effectiveness was compared with LOLA and rifaximin, there was no significant difference between the three antiammonium measures (*P* = 0.08). In comparison to LOLA and rifaximin, lactulose recorded multiple gastrointestinal adverse effects that were not severe; this is similar to a previous report by Als-Nielsen B, in a systematic review [26]. It is important to note that our results can be unpowered because this was a pilot study with a hypothetical sample size calculation based on a formula for contrasting hypothesis of two proportions. We recognize that we could make a mistake because we finally include four different treatment groups. With this in mind, we calculate again the sample size, this time using the statistical program G Power 3.1.9.2 to compare proportions between four groups, using as main statistical test X2 considering the command “goodness of fit tests: contingency tables” with a priori effect size of 0.40, alpha error of 0.05, statistical power of 80% (1-*β* = 0.80), and 4 degrees of freedom, obtaining a total sample size of 75 patients. However, if we increase the statistical power to 95% (1-*β* = 0.95), the total sample size increases to 117 patients. We consider that future clinical studies must be conducted to validate our findings.

In addition to a lower incidence in patients who received primary prophylaxis with some antiammonium therapy, it is noteworthy that in patients who did develop OHE the severity of the clinical HE determined by West-Haven scale was significantly lower in those who received some antiammonium drug, in comparison with the placebo group.

Bacterial infections, such as SBP, are recognized as a factor with a dominant role as a risk factor associated with the development of episodic HE [24]. However, in our study, very few patients (only 3) developed SBP, which explains the fact that, in the univariate analysis, this variable did not behave as a risk factor associated with development of HE. Interestingly, none of them were in the rifaximin group; therefore, this suggests the importance of designing specific clinical trials to validate if rifaximin can be an effective prophylactic therapy not only in avoiding the development of HE, but also in preventing the development of SBP in cirrhotic patients with AVB; if this finding is confirmed by future studies, it could be the most cost-effective strategy in this specific clinical scenario. Some previous studies suggest that rifaximin has an important role in regulating the intestinal microbiota [2, 27–29]. A recent meta-analysis, which included five studies with 555 patients, comparing rifaximin (295 patients) with systemic antibiotics (260 patients), found a potential protective effect of rifaximin (OR for SBP was 0.34; 95% CI 0.11-0.99; P < 0.05), with the advantage that rifaximin is a nonabsorbable drug compared to systemic antibiotics [30].

In our study, the recurrence of the AVB was the most important factor associated with the risk of developing OHE. Patients presenting with an episode of AVB have a risk higher than 60% of recurrence within the next year [25]. After the recurrence of the AVB, decompensated cirrhosis (Child B or C) was in our study the second most important factor which contributed to development of OHE. Similarly, Rattanasupar A et al. found that main risk factors for developing HE after an AVB were as follows: being Child C class, serum potassium < 3.5 mmole/L, leucocytes count > 10,000 cells/mm3, and hemoglobin level < 8 g/dL. Also, they found that cirrhotic patients with AVB who developed HE had high morbidity and mortality rates [31]. Based on the logistic regression model, our study suggests the introduction of any antiammonium drug could be a protective factor to prevent the development of OHE. However, this fact was not confirmed by the Cox regression models, maybe because of a small sample size in our study.

A limitation of our clinical trial is that we did not include patients with severe AVB who were hemodynamically unstable or who required orotracheal intubation at admission; it can compromise the external validity of our findings, which would be recommendable to perform new clinical trials to address the impact of primary prophylaxis with antiammonium drugs in this specific clinical context. Other interesting studies in hemodynamically unstable patients would be those conducted in “real life cohorts”.

Another important limitation of our study is that although it was a double-blind clinical trial, the blinding was imperfect because lactulose exerts a cathartic effect difficult to go unnoticed. In fact, the main adverse effects registered in our patients were gastrointestinal and were present in the group receiving lactulose.

## 5. Conclusions

In conclusion, our study shows that early primary prophylaxis with antiammonium drugs, particularly LOLA and rifaximin, seems to be a promising clinical strategy, effective and safe to avoid the development of OHE in cirrhotic patients with AVB. The most important risk factor associated with the development of OHE was the recurrence of the AVB.

## Figures and Tables

**Table 1:** Basal characteristics of patients.

**Characteristic**	**Lactulose**	**LOLA**	**Rifaximin**	**Placebo**	***P***
	**n = 22**	**n = 22**	**n = 21**	**n = 22**	
**Age (years)**	50.1 ± 11.3	54.3 + 7.7	53.0 + 10.9	49.3 ± 9.5	0.31

**Male gender, n (**%**)**	14 (63.6)	14 (63.6)	10 (47.6)	17 (77.3)	0.25

**CHILD A/B/C**	7/10/5	7/13/2	9/10/2	5/15/2	0.54

**Units of red cells concentrates**	1.1 ± 1.4	1.4 + 1.2	1.0 + 1.1	1.4 ± 1.1	0.51

**Cause of cirrhosis (n)**					
** Alcohol**	8	11	9	11	0.65
** Hepatitis C**	6	4	3	4	
** NASH**	4	7	5	4	
** Other**	4	0	4	3	

**MAP (mmHg)**	70.4 ± 10.3	68.4 ± 11.6	67.8 ± 10.3	65.7 ± 10.5	0.50

**Cardiac frequency (beats/min)**	90.9 ± 15.6	95.4 ± 12.3	88.8 ± 7.8	94.9 ± 10.4	0.47

**Time before endoscopy (hours)**	8.0 ± 1.9	7.0 ± 1.0	7.6 ± 1.6	7.0 ± 1.0	0.07

**Urea (mg/dl)**	41.4 ± 20.6	39.5 ± 18.6	41.9 ± 22.2	44.8 ± 22.2	0.86

**Creatinine (mg/dl)**	0.92 ± 0.26	0.81 ± 0.18	0.83 ± 0.21	0.93 ± 0.22	0.20

**Sodium (mEq/L)**	137.6 ± 3.7	138.2 ± 4.6	138.5 ± 3.1	136.6 ± 3.7	0.39

**Potassium (mEq/L)**	4.0 ± 0.5	3.9 ± 0.5	4.0 ± 0.5	3.9 ± 0.4	0.62

**Chlorine (mEq/L)**	104.9 ± 4.0	101.9 ± 4.2	103.2 ± 5.6	104.3 ± 6.2	0.24

**Albumin (mg/dl)**	3.0 ± 0.7	3.2 ± 0.5	3.0 ± 0.6	2.9 ± 0.7	0.47

**Bilirubin (mg/dl)**	1.7 ± 1.4	1.7 ± 1.2	1.7 ± 1.3	2.1 ± 1.8	0.69

**Hemoglobin (g/dl)**	9.9 ± 4.7	8.6 ± 2.9	8.8 ± 2.7	8.3 ± 2.7	0.46

**Hematocrit (**%**)**	30.1 ± 14.6	26.3 ± 8.3	26.7 ± 7.9	25.7 ± 7.9	0.50

**Platelets (10** ^**9**^ **cells/mcl)**	130.6 ± 95.3	107.3 ± 34.4	131.1 ± 69.7	127.5 ± 71.9	0.65

**Leucocytes (10** ^**3**^ **cells/mcl)**	8.1 ± 5.4	7.6 ± 2.8	6.8 ± 2.6	9.0 ± 5.1	0.39

**Neutrophils (10** ^**3**^ **cells/mcl)**	6.0 ± 4.0	5.7 ± 2.3	5.2 ± 2.3	7.0 ± 4.6	0.41

**Prothrombin time (sec)**	17.3 ± 5.9	15.6 ± 3.9	17.8 ± 10.7	16.7 ± 4.0	0.73

**INR**	1.5 ± 0.5	1.3 ± 0.4	1.5 ± 0.9	1.4 ± 0.3	0.64

**Variceal bleeding source** **Esophageal/Gastric (n)**	19/3	20/2	16/5	20/2	0.46

**Rebleeding, n (**%**)**	3 (13.6)	7 (31.8)	7 (33.3)	5 (22.7)	0.39

INR: international normalized ratio; LOLA: L-ornithine L-aspartate; MAP: mean arterial pressure; NASH: nonalcoholic steatohepatitis.

Statistical significance: *P* ≤ 0.05.

**Table 2:** Comparison between the characteristics of patients who develop and those who did not develop encephalopathy after acute variceal bleeding.

**Characteristic**	**With HE**	**Without HE**	***P***
	**(n = 59) **	**(n = 28)**	
**Age (years)**	50.8 ± 10.4	53.4 ± 8.9	0.25

**Male gender, n (**%**)**	35 (59.3)	20 (71.4)	0.27

**CHILD A/B/C**	24/29/6	4/19/5	0.03

**Units of red cells concentrates**	1.1 ± 1.1	1.5 ± 1.3	0.15

**Cause of cirrhosis (n)**			
** Alcohol**	24	15	0.48
** Hepatitis C**	14	3	
** NASH**	14	6	
** Other**	7	4	

**MAP (mmHg)**	69.6 ± 10.2	64.1 ± 10.8	0.02

**Cardiac frequency (beats/min)**	91.2 ± 12.6	95.3 ± 10.5	0.12

**Time before endoscopy (hours)**	7.6 ± 1.6	6.9 ± 1.1	0.02

**Urea (mg/dl)**	39.3 ± 19.4	47.4 ± 22.5	0.09

**Creatinine (mg/dl)**	0.87 ± 0.21	0.88 ± 0.22	0.75

**Sodium (mEq/L)**	137.9 ± 3.3	136.8 ± 4.7	0.22

**Potassium (mEq/L)**	4.0 ± 0.4	3.9 ± 0.6	0.75

**Chlorine (mEq/L)**	103.3 ± 4.5	103.3 ± 6.1	0.99

**Albumin (mg/dl)**	3.2 ± 0.5	2.7 ± 0.7	<0.0001

**Bilirubin (mg/dl)**	1.6 ± 1.3	2.1 ± 1.5	0.09

**Hemoglobin (g/dl)**	8.7 ± 3.1	9.0 ± 3.7	0.74

**Hematocrit (**%**)**	26.5 ± 9.1	27.8 ± 11.3	0.59

**Platelets (10** ^**9**^ **cel/mcl)**	120.1 ± 69.7	128.4 ± 65.9	0.60

**Leucocytes (10** ^**3**^ **cel/mcl)**	7.3 ± 3.7	8.7 ± 4.8	0.14

**Neutrophils (10** ^**3**^ **cells/mcl)**	5.4 ± 2.8	6.7 ± 4.4	0.16

**Prothrombin time (sec)**	15.4 ± 3.6	19.3 ± 9.3	0.03

**INR**	1.3 ± 0.3	1.6 ± 0.8	0.03

**Variceal bleeding source** **Esophageal/Gastric (n)**	50/9	25/3	0.74

HE: hepatic encephalopathy; INR: international normalized ratio; MAP: mean arterial pressure; NASH: nonalcoholic steatohepatitis.

Statistical significance: *P* ≤ 0.05.

**Table 3:** Factors related to the development of hepatic encephalopathy in patients with cirrhosis after an acute episode of variceal bleeding. Univariate analysis.

**Characteristic**	**Without HE**	**With HE**	**OR (95**%** CI)**	***P***
	**(n = 59)**	**(n = 28)**		
**Rebleeding, n (**%**)**	7 (11.9)	15 (53.6)	8.6 (2.9 - 25.3)^*∗*^	<0.0001

**Decompensated cirrhosis CHILD B/C, n (**%**)**	35 (59.3)	24 (85.7)	4.1 (1.3 - 13.4)^*∗*^	0.01

**SBP development, n (**%**)**	2 (3.4)	1 (3.6)	1.1 (0.09 - 12.2)	1.00

**Primary prophylaxis with any anti-ammonium drug, n (**%**)**	49 (83%)	16 (57.1%)	0.2 (0.09 - 0.7)^*∗∗*^	0.009

**Gastric variceal bleeding source, n (**%**)**	9 (15.3%)	3 (10.7%)	0.7 (0.2 - 2.7)	0.74

CI: confidence interval; HE: hepatic encephalopathy; OR: odds ratio; SBP: spontaneous bacterial peritonitis.

Statistical significance: *P* ≤ 0.05. ^*∗*^Risk factor. ^*∗∗*^Protective factor.

**Table 4:** Factors related to the development of hepatic encephalopathy in patients with cirrhosis after an acute episode of variceal bleeding. Multivariate analysis by binary logistic regression.

**Characteristic**	**OR (95**%** CI)**	***P***
**Recurrence of bleeding (yes)**	12.1 (3.5 - 42.5)^*∗*^	<0.0001

**Decompensated cirrhosis (CHILD B ó C)**	3.0 (1.0 - 15.1)^*∗*^	0.05

**Primary prophylaxis with any anti-ammonium drug**	0.2 (0.05 - 0.6)^*∗∗*^	0.006

CI: confidence interval; OR: odds ratio.

Statistical significance: *P*≤ 0.05. ^*∗*^Risk factor. ^*∗∗*^Protective factor.

**Table 5:** Factors related to the development of hepatic encephalopathy in patients with cirrhosis after an acute episode of variceal bleeding. Multivariate analysis by Cox regression models.

**MODEL 1**		

**Characteristic**	**OR (95**%** CI)**	***P***

**Recurrence of bleeding (yes)**	1.9 (0.8 – 4.5)	0.17

**Decompensated cirrhosis (Child-Pugh B ** **or** ** C)**	1.0 (0.4 – 3.0)	0.96

**Primary prophylaxis with any anti-ammonium drug**	0.6 (0.2 – 1.5)	0.28

**MODEL 2**		

**Characteristic**	**OR (95**%** CI)**	***P***

**Recurrence of bleeding (yes)**	1.6 (0.6 – 4.4)	0.39

**Cirrhosis (Child-Pugh A)**	-	0.50
** (i) Child-Pugh B**	0.8 (0.2 – 2.8)	0.76
** (ii) Child-Pugh C**	1.7 (0.4 – 7.3)	0.46

**Group of Treatment (Placebo)**	-	0.69
** (i) LOLA**	0.6 (0.2 – 2.3)	0.47
** (ii) Lactulose**	0.5 (0.2 – 1.7)	0.26
** (iii) Rifaximin**	0.6 (0.2 – 2.0)	0.41

CI: confidence interval; LOLA: L-ornithine L-aspartate; OR: odds ratio.

Statistical significance: *P*≤ 0.05.

## Data Availability

Additional data used to support the findings of this study are available from the corresponding author upon request.

## Abbreviations

**AASLD**: ** American Association for Study of Liver Disease**
**AVB**: ** Acute variceal bleeding**
**CCF**: Critical flicker frequency
**EASL**: European Association for the Study of the Liver
**HE**: Hepatic encephalopathy
**INR**: International normalized ratio
**LOLA**: L-Ornithine L-aspartate
**MHE**: Minimal hepatic encephalopathy
**NASH**: Nonalcoholic steatohepatitis
**OHE**: Overt hepatic encephalopathy
**PHES**: Psychometric Hepatic Encephalopathy Score
**PMN**: Polymorphonuclear cells
**SBP**: Spontaneous bacterial peritonitis.
